# Polyoxometalate-Doped Hole Transport Layer to Boost Performance of MaPbI_3_-Based Inverted-Type Perovskite Solar Cells

**DOI:** 10.1021/acsomega.4c01242

**Published:** 2025-02-14

**Authors:** Sumeyra Buyukcelebi, Mehmet Kazici, Yasemin Torlak, Mahmut Kus, Mustafa Ersoz

**Affiliations:** † Advanced Technology Research and Application Center, Selcuk University, Konya 42075, Turkey; ‡ Engineering Faculty, Department of Electrical & Electronics Engineering, Siirt University, Siirt 56100, Turkey; § Cal Vocational High School, Pamukkale University, Denizli 20160, Turkey; ∥ Department of Chemical Engineering, Konya Technical University, Konya 42250, Turkey; ⊥ Department of Chemistry, Selcuk University, Konya 42250, Turkey

## Abstract

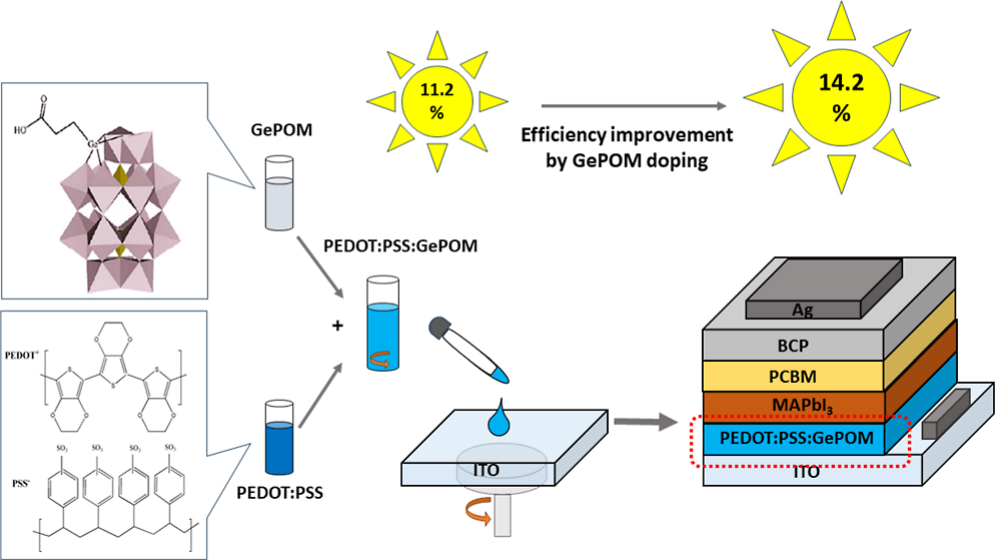

This study delves into the examination of the efficiency, stability, and repeatability of perovskite solar cells (PSCs), a focal point in contemporary photovoltaic (PV) technologies. The aim is to address the challenges encountered in PSCs. To achieve this goal, Ge-doped polyoxometalate, a structure of significance in recent molecular electronics, was employed as a dopant in the hole transport layer (HTL). The study investigated alterations in the conductivity, improvements in efficiency, and changes in PV parameters. The utilization of PEDOT/PSS doped with a maximum of 2% GePOM resulted in an average efficiency increase of 27% in PSCs compared with the reference. Moreover, enhancements in stability and repeatability were also noted. Comparatively, the reference PSC operated at an efficiency of 11.18%, while PSCs incorporating 2% GePOM into PEDOT/PSS as the HTL exhibited a notable increase in the efficiency, reaching 14.22%. Furthermore, the champion device exhibited an observed fill factor value of 0.74, a short-circuit current density (*J*_sc_) value of 19.78 mA/cm^2^, and an open-circuit voltage (*V*_oc_) value of 0.98 V. Consequently, noteworthy enhancements have been noticed in the PV parameters of PSCs with the introduction of GePOM doping.

## Introduction

Since the first research paper on perovskite solar cells (PSCs) was published in 2009, many different research groups and industries have grown increasingly intrigued by PSCs, primarily due to the remarkable improvement in their power conversion efficiency (PCE), which increased from 3.8% to 26.1.^1,2^ The impressive combination of superior performance and the use of solution-based processes in PSCs plays a pivotal role in shaping the future of photovoltaic (PV) technology.^3^ In 2009, Miyasaka and his colleagues pioneered the use of perovskite materials for PV applications, achieving an initial PCE of only 3.8%.^1^ However, it was realized that perovskite materials rapidly degraded in the liquid electrolyte; therefore, the search for suitable production methods commenced.^4^ In 2012, to address the issue of instability problem of perovskite materials in liquid electrolytes, solid-state device configurations were developed for PSCs based on thin films.^5,6^ The PCEs have achieved about 10% by improving device stability. At the present time, Park and his co-authors obtained world record by using anion engineering concept for PSCs with PCEs of 26.1%.^2^ To enhance the stability and PCEs of PSCs, each stage of the fabrication process holds crucial significance. For single-junction PSCs, there are two distinct device types: conventional and inverted. The primary difference between these two devices is the direction of the electrons and holes. In conventional PSCs, electrons migrate toward the back electrode, whereas in inverted PSCs, they move toward the top electrode. Additionally, in the conventional device structure, holes are collected in the top electrode, while in the inverted device structure, they are gathered at the back electrode. The electron and hole selective layers employed in both conventional and inverted device structures significantly influence the PV performance of PSCs.^7,8^ Poly(3,4-ethylenedioxythiophene)/polystyrenesulfonate (PEDOT/PSS) widely used organic hole transport layer (HTL) material for inverted-type PSCs.^9−12^ Many studies have been carried out to enhance the PV performance of the inverted-type PSCs by tailoring PEDOT: PSS.^13−15^ Unfortunately, the limited electron-blocking efficiency, acid corrosiveness, and hygroscopic characteristics of PEDOT/PSS make it an unsuitable HTM for use in devices.^14^ The easiest approach to resolve these obstacles is by doping PEDOT/PSS. By doping different materials to PEDOT/PSS such as DMSO,^16^ urea,^17^ metal oxides,^18^ dopamine,^19^ ionic liquid,^20^ and CuSCN,^21^ significant improvements have been achieved in both the PCEs and stability of MAPbI_3_-based PSCs. Duan and his colleagues showed that the perovskite films coated on PEDOT/PSS by doping some ammonium salts still had a good morphology within 20 days under atmospheric conditions, and they explained this by the ammonium salts preventing the damage caused by water to the perovskite layer.^18^

Recently, polyoxometalate (POM) materials, a type of nanosized metal–oxygen clusters, have been used as charge-selective layers in dye-sensitized,^22^ organic,^23^ and PSC^24^ applications. POMs possess a range of advantageous properties, including excellent thermal stability, a substantial molecular size, and nontoxicity.^25^ In PV applications, Dawson- and Keggin-type POMs find extensive use. In third-generation PV technologies, Dawson-type POMs serve as HTLs, whereas Keggin-type POMs function as electron transporting layers.^23,26^ Fang et al. achieved significantly higher efficiency values in organic solar cells by employing Dawson-type materials as HTLs instead of PEDOT/PSS.^27^ A novel material based on POM was employed as an additive into 2,2′,7,7′-tetrakis(*N*,*N*-dimethoxyphenyl-amine)-9,9′-spirobifluorene (Spiro-OMeTAD), which is one of the most widely utilized as HTL in conventional type PSCs, to improve both PCEs and stability.^28^ Pristine organic HTLs have been observed to significantly limit the PV performance of PSCs. Nevertheless, when HTLs are doped using appropriate strategies, there is a noticeable improvement in PCEs.^29^

We have not reached any paper related to doping of Dawson-type POMs in the PEDOT/PSS layer for improving the efficiency as well as stability of PSCs. In the present study, we have investigated Dawson-type GePOM ([α_2_-P_2_W_17_O_61_(HOOC(CH_2_)_2_Ge)]^7–^) as a dopant into PEDOT/PSS for the fabrication of MAPbI_3_-based inverted-type PSCs under high moisture (40–60%) and room conditions (∼25 °C) for the first time. The unique properties of this additive material led to a significant improvement in the conductivity of GePOM-doped PEDOT/PSS.

The PV results indicate that GePOM-doped devices have demonstrated improvements in both short-circuit current density (*J*_sc_) and fill factor (FF), owing to enhanced hole extraction and charge separation. The concentration of GePOM doping was meticulously optimized, resulting in the enhanced performance in terms of both PCEs and stability of the PSCs.

## Experimental Section

### Synthesis of GePOM

**Figure 1:**
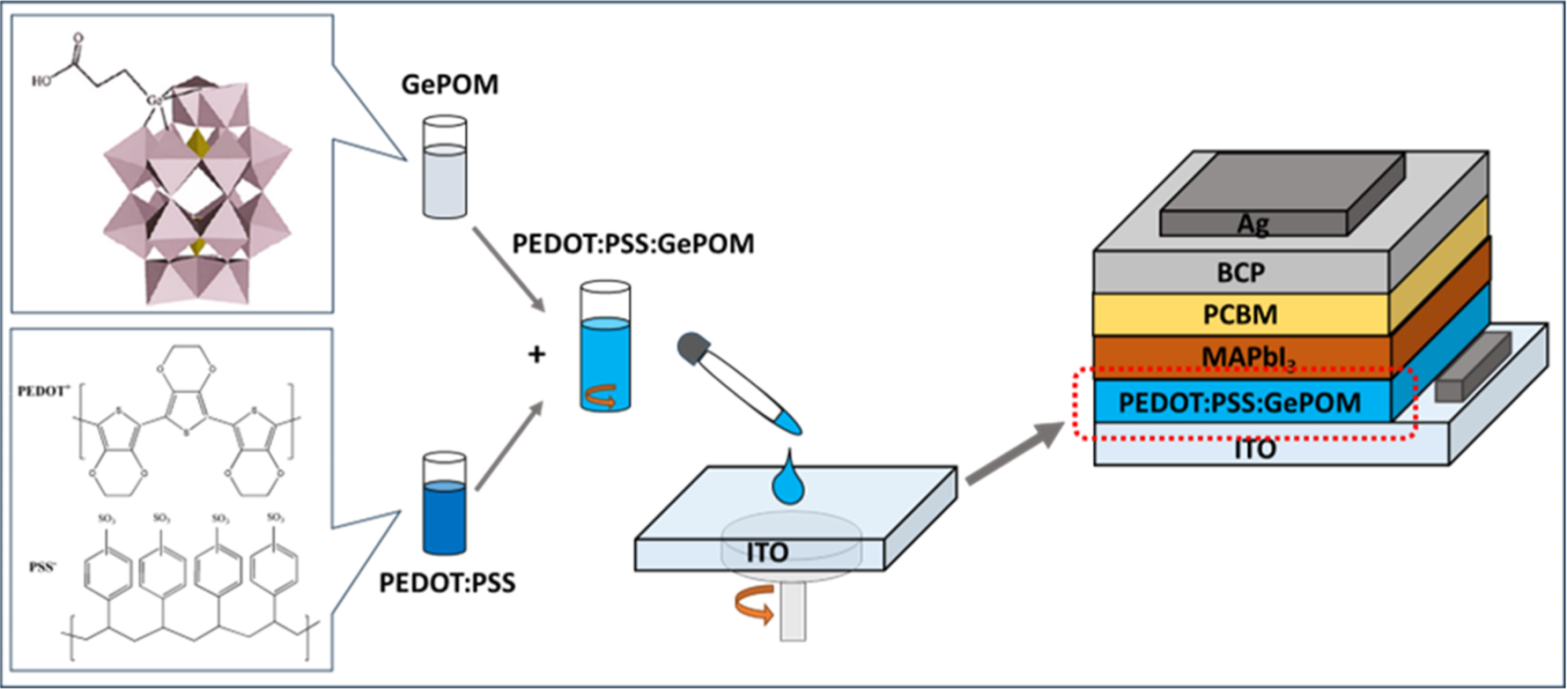
Device configuration of the PSCs and schematic illustration of the experimental setup to prepare the PEDOT/PSS:GePOM solution (GePOM structure reproduced with permission Copyright 2011, American Chemical Society^30^).

The [α_2_-P_2_W_17_O_61_(HOOC(CH_2_)_2_Ge)]^7–^ was synthesized according to previous work.^30^ Cl_3_Ge-(CH_2_)_2_COOH (Sigma-Aldrich, 1.0 mmol) was dissolved with magnetic stirring at room temperature in 30 mL of ultrapure water. K_10_[a_2_-P_2_W_17_O_61_]_3_ 24H_2_O (Sigma-Aldrich, 1.0 mmol) was added with stirring to this clear solution. After the solution was stirred for 30 min, Me_2_NH_2_CI (Sigma-Aldrich, 67.5 mmol) was added. The white precipitate was filtered, washed with ethanol and diethyl ether solution (three times of 50 mL), and vacuum-dried. The molecule of [α_2_-P_2_W_17_O_61_(HOOC(CH_2_)_2_Ge)]^7–^ polyoxoanion (GePOM) was obtained with a yield of 50%. The molecular structure of GePOM is shown in Figure 1.

### Device Fabrication

PSCs were prepared with an ITO/PEDOT/PSS/MAPbI_3_/PCBM/BCP/Ag-based inverted structure. ITO-coated glasses (Japan, 6–7 Ω/sq resistance, polished grade) were cleaned with acetone and isopropanol in ultrasonic bath for 30 min each and dried by spraying nitrogen gas. Before spin coating, cleaned ITO glasses were exposed to oxygen plasma (Germany, diener-femto) for 10 min. GePOM were dissolved in extra pure water at concentration of 10 mg/mL. After that, different volume percentages of GePOM (0%, 1%, 2%, and 3%) were added into the PEDOT/PSS (Heraeus, AL4083) solution. Soon after the oxygen plasma treatment, the PEDOT/PSS and doped solutions were spin-coated on clean ITO glasses at 2000 rpm for 1 min and annealed on a hot plate at 140 °C for 10 min. To prepare the perovskite solution, PbI_2_ (Sigma-Aldrich, 1.3 M) and CH_3_NH_3_I (Lumtec, 1.54 M) were dissolved in 2.5 mL of g-butyrolactone (GBL) and stirred with the magnetic stirrer at 50 °C for 24 h. The solution was filtered with 0.45 μm PTFE before coating. Perovskite solution was deposited on the PEDOT: PSS layer at 5000 rpm for 40 s. Toluene (antisolvent, 80 μL) was dripped over the spinning substrate during spin coating at the last 10 s. The films were annealed at 100 °C for 20 min. PCBM (Lumtec) solution (20 mg/mL in chlorobenzene) was spin coated onto the perovskite-coated substrates at 2000 rpm for 35 s. After that, a BCP (Lumtec) solution (0.5 mg/mL in ethanol) was coated at 4000 rpm for 45 s. Finally, the 110 nm Ag electrode was thermally deposited. Each concept was completed under the same condition. The schematic device structure of the fabricated PSC and molecular structures of GePOM and PEDOT/PSS are illustrated in Figure 1.

### Characterization and Measurements

Photoluminescence (PL) spectra were recorded using a Hitachi, F-7000 fluorescence spectrophotometer at the same sample position. Fourier transform infrared (FT-IR) spectrophotometer spectra were obtained on a Bruker Tensor 27 spectrometer. Surface morphology was investigated with an NT-MDT Ntegra Solaris atomic force microscope (AFM) using the NSG03 silicon probe with a resonant frequency of 47–150 kHz. The measurements were scanned in the tapping mode. Film thicknesses were measured with an AEP Nanomap-500LS stylus profilometer. The current and voltage (*I*–*V*) measurements of PSCs were characterized using a Keithley 2400 source-meter under ATLAS (AM 1.5G filter, 100 mW/cm^2^) solar simulator in glovebox. The photoactive area of the solar cells was defined as 0.023 cm^2^ with a mask. For stability measurements of PSCs, all solar cells were stored in a nitrogen atmosphere in the glovebox. The incident photon to electron conversion efficiency (IPCE) spectra were taken with a model Newport QE/IPCE measurement system by using a 300 W Xe lamp. A standard silicon solar cell was used to calibrate the light intensity and operated in the measuring range of 350–850 nm in 5 nm steps.

## Results and Discussion

PEDOT possesses a positively charged conjugated structure, along with conductive and hydrophobic properties, while PSS exhibits a negatively charged nonconjugated structure, nonconductive properties, and hydrophilic characteristics. The formation of PEDOT/PSS results from the strong Coulombic interactions between PEDOT and PSS components.^13^ As a consequence, the interaction between PEDOT and PSS can be weakened through a doping strategy, and enhancing the linear structure of PEDOT chains can improve its charge-carrying ability as a HTL.^31^

**Figure 2:**
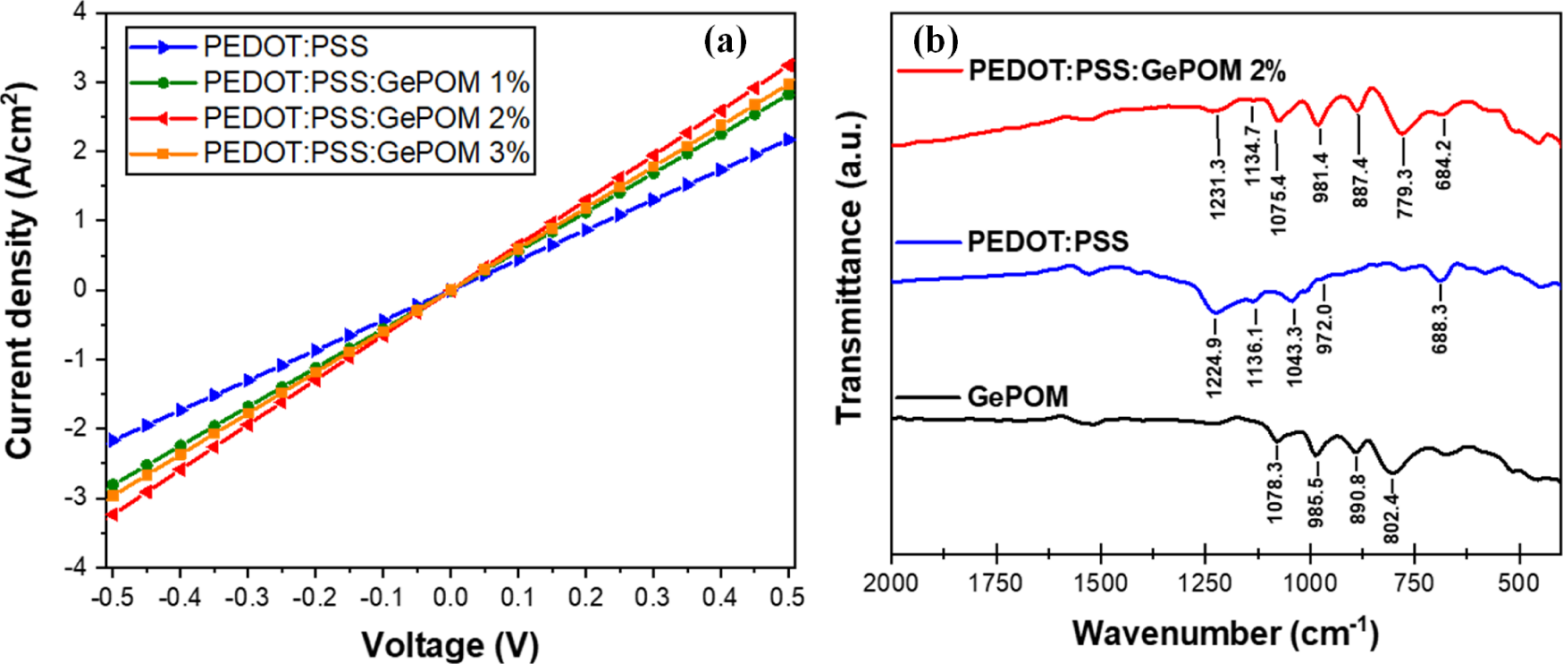
(a) Current density–voltage characteristics of different concentrations (1%, 2%, and 3%) of Ge-POM-doped and undoped PEDOT/PSS films and (b) FT-IR spectra of GePOM, pristine PEDOT/PSS, and the 2% GePOM-doped PEDOT/PSS films.

The electrical conductivity of PEDOT/PSS plays a crucial role in the performance of PV devices. Improving the electrical conductivity of PEDOT/PSS, in particular, may lead to a decrease in series resistance (*R*_s_), resulting in a boost in the PV performance of PSCs due to efficient charge transport between HTL and perovskite. Doped and undoped PEDOT/PSS solutions were coated on ITO electrodes to better understand the impact of Ge-POM on the electrical conductivity of PEDOT/PSS. The current density–voltage (*J–V*) characteristics of ITO/HTL/Ag devices were investigated. Figure 2 depicts current density–voltage measurements for doped and undoped PEDOT/PSS films (with the ITO/HTL/Ag configuration).

To gain deeper insight into the impact of the electrical conductivity on the HTL, we investigated various concentration ratios of Ge-POM into PEDOT/PSS, as depicted in Figure 1a. The electrical conductivity “**σ**_***E***_” of doped and undoped thin films can be calculated using the equation below^32,33^
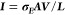
1

In this context, “***L***” represents the thickness of the films, which is approximately 50 nm. “***A***” stands for the active area, measured at 0.023 cm^2^. “***V***” denotes the voltage, and “***I***” represents the current. The slope of the *J–V* graph in Figure 2a can be utilized to compare the electrical conductivities of doped and undoped HTLs. When the slope of the *J–V* graph increases, it indicates a significant enhancement in the electrical conductivity of the thin film, as described by eq 1. Figure 2a clearly illustrates that the doped HTL with a 2% concentration exhibits the highest slope value, while the undoped HTL displays the lowest slope value. The electrical conductivities of undoped and doped HTL (with a 2% concentration) were calculated to be 2.17 × 10^–5^ and 3.24 × 10^–5^ S/cm, respectively. To strengthen the validity of the electrical conductivity measurements and to more clearly understand the effect of GePOM ratios on electrical conductivity, some statistical measurements were performed (Figure S1). It was observed that the electrical conductivity of the Ge-POM doped HTL is substantially higher than that of the pristine HTL. The improvement in the electrical conductivity of the doped HTL might be attributed to the interaction between the negatively charged Ge-POM molecules and the positively charged PEDOT molecules, which in turn promotes a more linear structure in the PEDOT chains.^34^ We would like to emphasize here that the difference between the HOMO energy levels for PEDOT/PSS and GePOM is too large for both materials to affect each other in such a way as to change the energy level^35^ (see Figure S2).

FT-IR measurements were conducted to investigate the presence of Ge-POM in the PEDOT/PSS. FT-IR spectra within the range of 400–2000 cm^–1^ were utilized to analyze both the Ge-POM doped (2%) and undoped PEDOT/PSS. The FT-IR spectra of the thin films coated onto ITO substrates are presented in Figure 2b.

The characteristic peaks observed at 1224.9 and 1043.3 cm^–1^ in the spectrum of undoped PEDOT/PSS can be attributed to the asymmetric and symmetric S–O bonds in the thiophene ring.^36^ The peak at 1136.1 cm^–1^ corresponds to the stretching of the C–O–C bonds within the aromatic ring, while the peaks at 972.0 and 688.3 cm^–1^ represent the stretching of the C–S bonds within the thiophene ring.^37^ The characteristic vibrational bands associated with the Dawson structure can be observed in the GePOM FT-IR spectra within the range of 700 and 1100 cm^–1^^30^^,^.^38^ P–O, W–O, W–Oe–W (edge-sharing oxygen atoms), and W–Oc–W (corner-sharing oxygen atoms) bands are assigned to the characteristic peaks observed at 1078.3, 985.5, 890.8, and 802.4 cm^–1^, respectively. The FT-IR spectrum of the GePOM-doped (2%) PEDOT/PSS thin film exhibits the same characteristic vibrational peaks, providing strong evidence for the presence of GePOM in the doped PEDOT/PSS thin film. We have indicated that the observation of slight shifts toward lower wavenumbers for some vibrations, such as P–O, W–O etc., may be attributed to chemical interactions between PEDOT/PSS and GePOM as well as the change of electronic environment by doping. Consequently, the FT-IR analysis of both doped and undoped HTLs confirms the presence of GePOM in the doped HTL.

**Figure 3:**
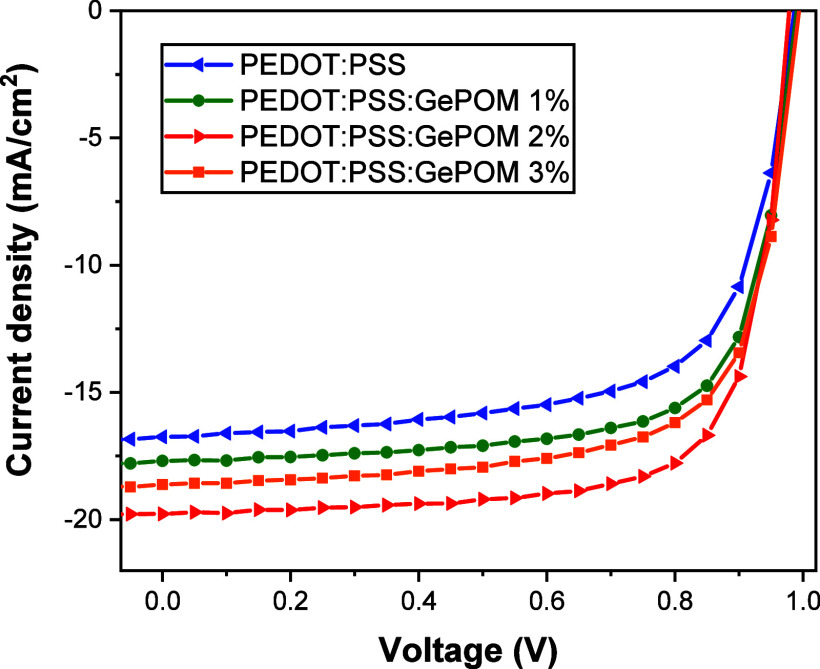
*J–V* curves of MAPbI3-based PSCs fabricated using GePOM-doped HTLs.

After investigating the beneficial effects and presence of GePOM as an additive on the HTL, we fabricated inverted-type PSCs based on MAPbI_3_ under high moisture (40–60%) and room conditions (∼25 °C). The *J–V* curves of the resulting PV devices are illustrated in Figure 3.

The undoped HTL, consisting of pristine PEDOT/PSS, served as the control device, exhibiting a short-circuit current density (*J*_sc_) of 16.75 mA/cm^2^ and an open-circuit voltage (*V*_oc_) of 0.99 V. The PCE was calculated to be 11.18%, with a FF of 0.68%. When GePOM was introduced as a dopant at a 1% concentration in the HTL, the resulting devices achieved a *J*_sc_ of 17.69 mA/cm^2^, *V*_oc_ of 0.99 V, and FF of 0.72, leading to an improved PCE of 12.53%. For devices with a 2% GePOM-doped HTL, *J*_sc_ increased to 19.78 mA/cm^2^, *V*_oc_ remained at 0.98 V, and FF improved to 0.74, resulting in a significantly higher PCE of 14.22%. In the case of devices with a 3% GePOM-doped HTL, the *J*_sc_ reached 18.62 mA/cm^2^, *V*_oc_ remained at 0.99 V, and FF was 0.70, resulting in a PCE of 12.99%. The PV results for the fabricated devices are summarized in Table 1. Notably, the device employing a 2% GePOM-doped HTL demonstrated the highest performance among all tested configurations, achieving a PCE of 14.22%.

**Table 1:** Photovoltaic Parameters Extracted from *J–V* Curves in Figure 2

GePOM content [wt %]	*J*_SC_ (mA/cm^2^)		*V*_OC_ (V)		FF		resistance (Ω cm^2^)		PCE (%)	
	average	best	average	best	average	best	*R*_s_	*R*_sh_	average	best
0	16.05	16.75	0.98	0.99	0.67	0.68	12.73	662.3	10.55	11.18
1	17.71	17.69	0.97	0.99	0.69	0.72	11.92	978.5	11.89	12.53
2	19.28	19.78	0.98	0.98	0.72	0.74	8.85	1098.9	13.66	14.22
3	18.78	18.62	0.99	0.99	0.68	0.70	12.08	892.9	12.68	12.99

The champion device, incorporating GePOM (2%) as an additive in the HTL, exhibited a remarkable 27% improvement in PV performance compared to the control device. The PV results clearly indicate that the champion device achieved simultaneous enhancements in both the short-circuit current density (*J*_sc_) and FF by incorporating GePOM into the HTL. The values of series resistance (*R*_s_) and shunt resistance (*R*_sh_) can exert a significant influence on device performance in PSCs. As detailed in Table 1, we have provided *R*_s_ and *R*_sh_ values for both the control and the doped devices. It is worth noting that enhancing the electrical conductivity of PEDOT/PSS^39^ may lead to a reduction in series resistance losses, consequently resulting in an increase in the PCE.

As can be seen in Table 1, for the control and GePOM doped (2%) HTL devices, series resistance values (*R*_s_) were determined to be 12.73 and 8.85 Ω cm^2^, respectively. It is evident that an increase in *R*_s_ leads to a reduction in short-circuit current density. The enhanced electrical conductivity of the HTL, thanks to the GePOM additive, might be one of the contributing factors to the lower series resistance observed in the GePOM-doped HTL device.

**Figure 4:**
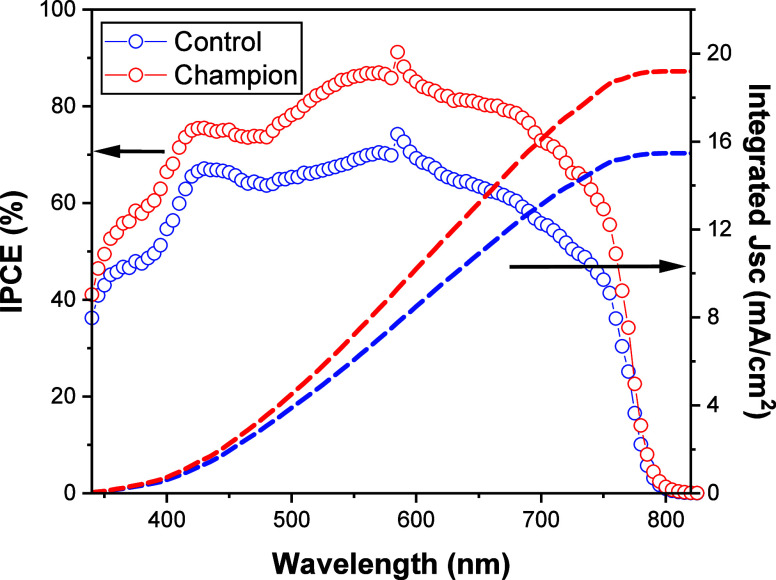
IPCE spectra and the integrated *J*_sc_ of the control and champion PSCs.

External quantum efficiency (EQE) is a critical parameter to determine the efficiency of solar cells including PSCs. The EQE spectra measurements were performed for the control and champion devices. The EQE spectra for fabricated PSCs are shown in Figure 4.

When used in the two different HTLs (PEDOT/PSS and GePOM-doped PEDOT/PSS), the PSCs exhibit drastically different light-harvesting properties, as shown by the EQE spectra in Figure 4. Notably, the champion device exhibits a prominent EQE peak at 600 nm, boasting a quantum efficiency of approximately 90%. In contrast, the control device displays a comparatively weaker EQE peak at the same wavelength with a quantum efficiency of around 70%. To verify the accuracy of the short-circuit current density (*J*_sc_) values obtained from the *J*–*V* graphs, we computed *J*_sc_ values from the EQE graph. The photocurrent densities for the control and champion devices, calculated from the EQE graph, were determined to be 15.47 and 19.19 mA/cm^2^, respectively. These EQE results confirm that the photocurrent densities derived from both *J–V* and EQE measurements are in good agreement.

**Figure 5:**
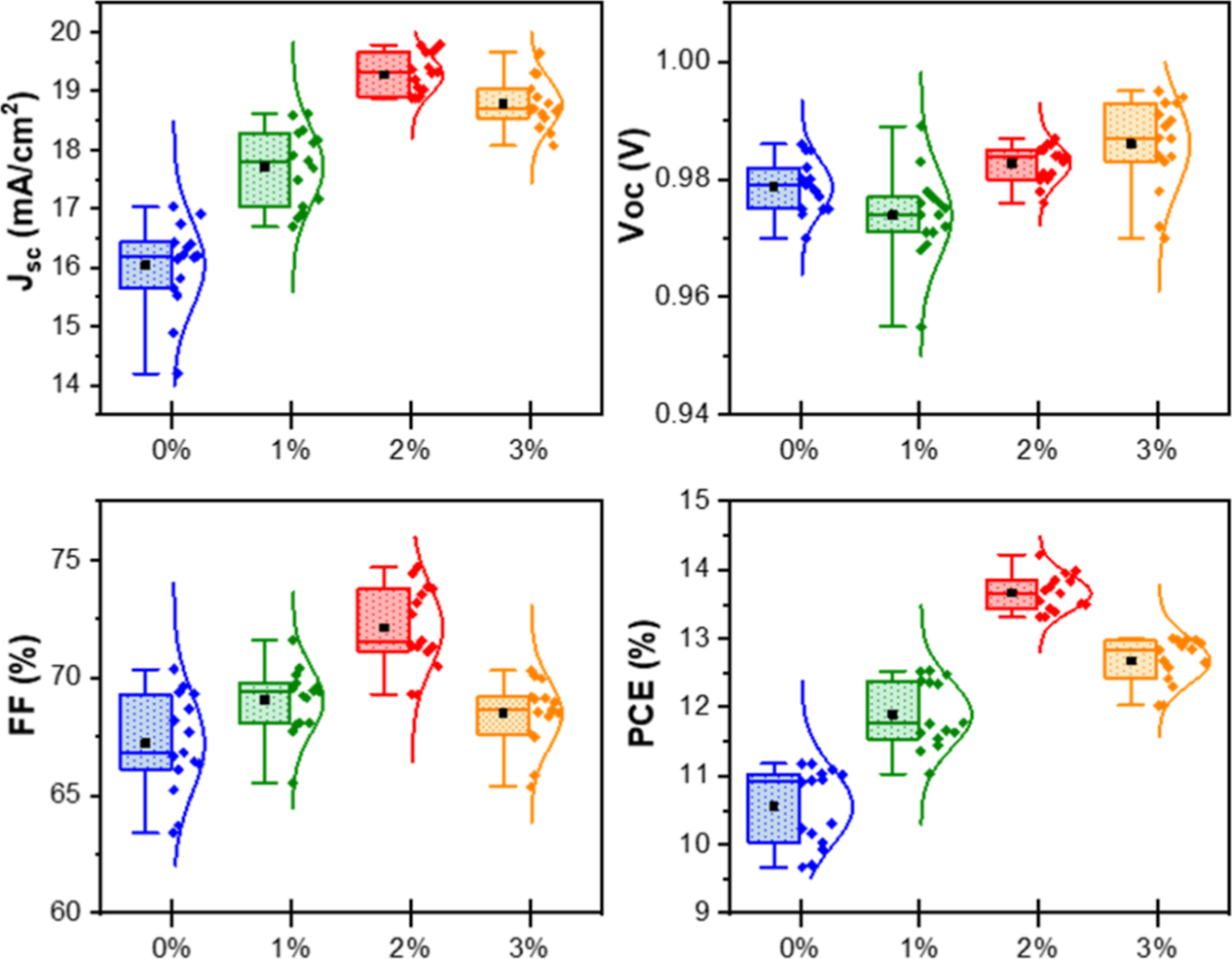
Statistic distribution of PV parameters of 60 (60) independent devices.

Statistic distribution of PV parameters of 60 (60) independent devices is depicted in Figure 4. It is obviously seen from Figure 5, while the *J*_sc_ values for the undoped HTLs (control devices) range from 14.20 to 17.04 mA/cm^2^, for the GePOM-doped HTLs (2%), values vary from 18.88 to 19.79 mA/cm^2^. Even when comparing the highest *J*_sc_ value (17.04 mA/cm^2^) for devices utilizing pristine PEDOT/PSS as the HTL with the lowest *J*_sc_ value (18.88 mA/cm^2^) in GePOM-doped devices, statistic distribution results show that the current density values have been enhanced by employing GePOM to the HTL. Another key PV parameter, the FF, has also demonstrated improvement. The highest FF values for the pristine and GePOM-doped HTL devices were 0.70 and 0.75, respectively. Additionally, the statistical analysis of PV parameters reveals that the open-circuit voltage (*V*_oc_) values, ranging from 0.97 to 0.99 V, remain approximately consistent across the fabricated devices.

**Figure 6:**
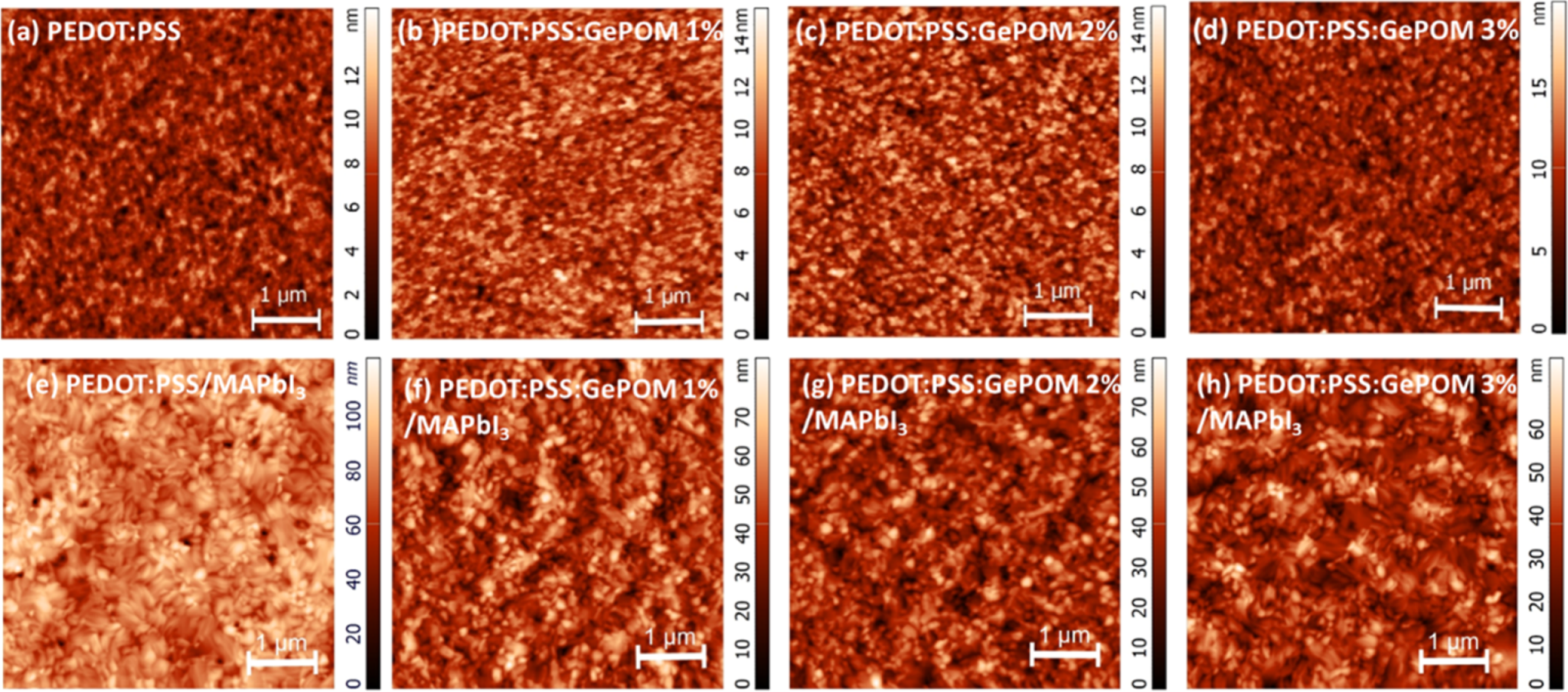
AFM height images for (a–e) PEDOT/PSS and perovskite on PEDOT/PSS, (b–f) PEDOT/PSS/GePOM (1%) and perovskite on PEDOT/PSS/GePOM (1%), (c–g) PEDOT/PSS/GePOM (2%) and perovskite on PEDOT/PSS/GePOM (2%), and (d–h) PEDOT/PSS/GePOM (3%) and perovskite on PEDOT/PSS/GePOM (3%).

The surface morphology of thin films in PSCs has a significant effect on PV performance. We have to indicate that the grain size characteristics of the SEM images are very similar in all cases (see Figure S3). Therefore, the only situation where we observed some differences in correlating the efficiency values with the film surfaces is the AFM data. For this reason, the explanation in this section is based on AFM data. We examined the surface morphology of both the HTLs and the perovskite layers deposited on the HTLs using AFM. The AFM height images for the HTLs and perovskite layers are illustrated in Figure 6.

**Figure 7:**
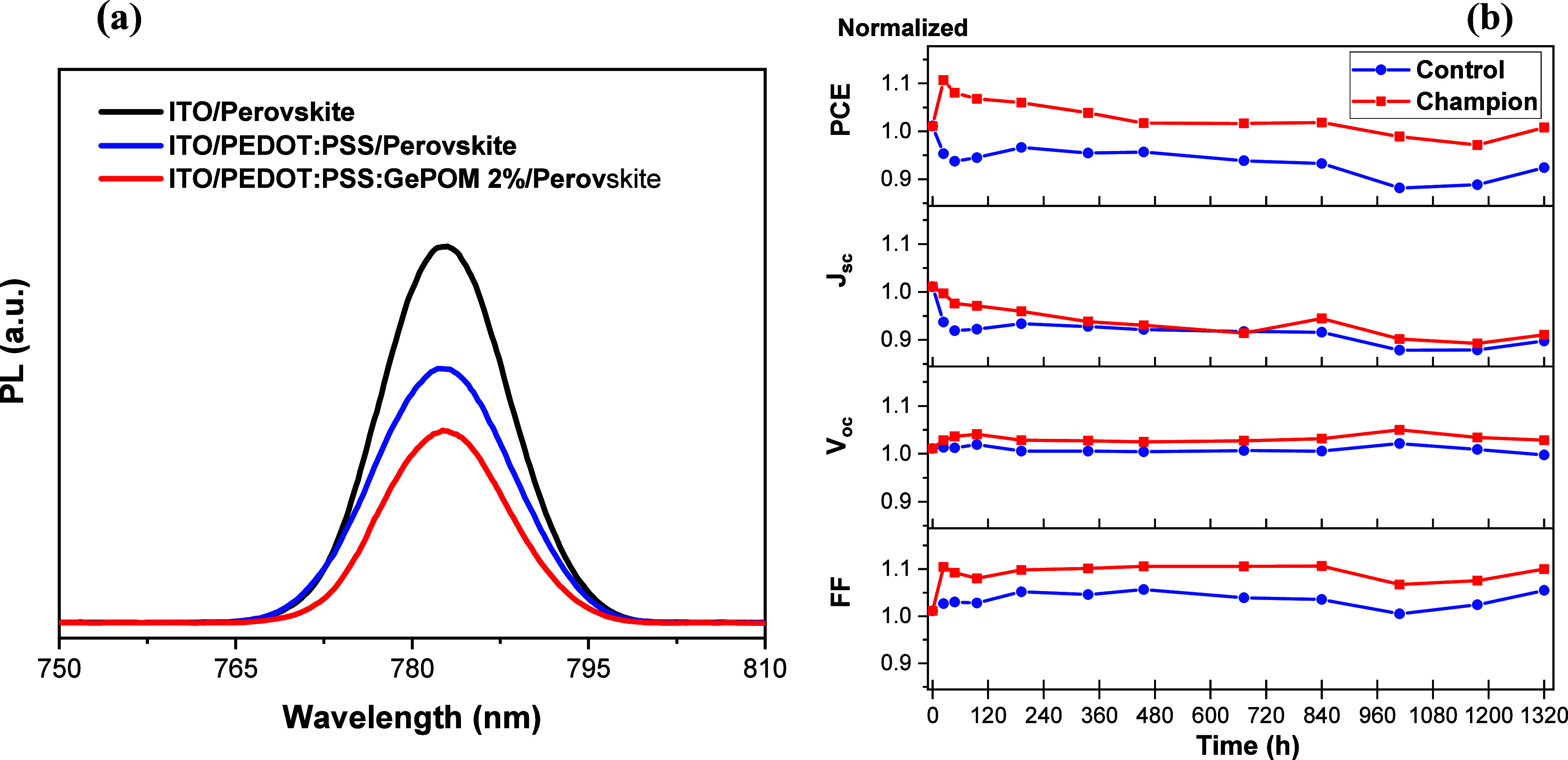
(a) Steady-state PL spectra of perovskite layers on ITO, PEDOT/PSS, and PEDOT/PSS/GePOM (2%). (b) Normalized PCE and PV parameters of PSCs based for control (blue) and champion (red) devices.

The root-mean-square (RMS) roughness values of PEDOT/PSS, PEDOT/PSS/GePOM (1%), PEDOT/PSS/GePOM (2%), and PEDOT/PSS/GePOM (3%) are 1.621, 1.793, 2.039, and 2.202 nm, respectively. The surface roughness measurements indicate that as the doping concentrations of GePOM increase, the HTLs become rougher. The surface roughness of HTLs plays a significant role in influencing charge carrier formation in MAPbI_3_-based PSCs, which is closely related to exciton generation, lifetime, and dissociation. Modifying the surface roughness of HTL thin films may result in enhancements in the short- *J*_sc_ values of PSCs.^40^ The obtained PV results confirm the effectiveness of this approach with *J*_sc_ values for the control and champion devices measuring 16.75 and 19.78 mA/cm^2^, respectively. Another crucial aspect for improving the PV performance of PSCs is the surface roughness of the perovskite layer. The RMS roughnesses of perovskite layers on pristine and GePOM-doped HTLs (1%, 2%, and 3%) were measured as 11.69, 11.19, 10.36, and 9.88 nm, respectively. While there is a slight trend toward smoother perovskite surfaces as the GePOM concentration increases, the differences in roughness across the perovskite surfaces are relatively small. To further examine the HTL/perovskite interface, PL spectroscopic measurements were performed to analyze the quenching of perovskite on HTLs. The PL spectra of perovskite thin films deposited on ITO, pristine PEDOT/PSS, and GePOM-doped (2%) PEDOT/PSS are depicted in Figure 7a. Notably, prominent peaks at 783 nm corresponding to emissions from MAPbI_3_ thin films were observed in the PL measurements. The quenching effect can be clearly observed at the PEDOT/PSS/perovskite interface (blue line in Figure 7a), indicating charge transfer between PEDOT/PSS perovskite compared to ITO/perovskite. On the other hand, it can be clearly observed that the quenching effect is further enhanced by doping GePOM into PEDOT/PSS. The significant decrease in the intensity of the peak at 783 nm indicates a more favorable charge transfer. This suggests that it is easier to transfer of the photogenerated holes in perovskite to the GePOM-doped HTLs.^40,41^ In other words, the trend of *J*_sc_ in Table 1 may be explained by the exciton dissociation (PL quenching) at the HTL/perovskite interface increasing when the GePOM (2%) is utilized as an additive in PEDOT/PSS.

One of the most critical issues of mass production for PSCs is the stability. Therefore, for control and champion devices, stability measurements were performed in glovebox without encapsulation for 1320 h (55 days). Normalized stability measurements of PV parameters are illustrated in Figure 7b. After 1320 h, stability measurements revealed that the champion device had essentially no loss of PCE, while the control device had a loss of 8%. It was observed that GePOM doping into HTL enhances the stability of PSCs. As well-known POMs have hygroscopic properties, which means they can absorb and block moisture from reaching the perovskite layer, protecting it from degradation.^42^ On the other hand, perovskite materials contain mobile ionic species, such as lead ions (Pb^2+^), which can migrate within the perovskite layer. These migrations can cause structural defects and lead to hysteresis in the current–voltage characteristics of the PSCs. This idea is also supported by comparing the hysteresis behavior for both device structures. We observe that hysteresis effect is decreased by doping GePOM into the PEDOT/PSS layer (see Figure S4). In addition to the increase in the efficiency, the decrease in the level of hysteresis indicates a decrease in ionic migration. Ionic migration can also affect the stability of the perovskite/PEDOT/PSS interface, potentially leading to degradation. POMs doped in PEDOT/PSS may passivate surface states, which may prevent ion transfer which causes the degradation of the perovskite layer, resulting in improved stability. The most favorable pathways of the passivation mechanism can be explained as a chemical interaction. GePOM interacts chemically with uncoordinated Pb^2+^ ions, thereby filling the vacancies that cause ion migration most probably due to OH groups of POMs.^43^ By passivating surface defects, GePOM reduces nonradiative recombination centers, which helps protect the structural integrity of the perovskite layer and reduces ion mobility.

## Conclusions

In this work, the influence of incorporating GePOM into PEDOT/PSS as a HTL for PSCs has been investigated. MAPbI_3_-based inverted-type PSCs were fabricated under high moisture (40–60%) and room conditions (∼25 °C). This study revealed that the conductivity of PEDOT/PSS improved with the introduction of POM structure doping at certain ratios. Furthermore, the POMs demonstrated the ability to hinder ion migration through surface passivation, resulting in an increased stability. Ultimately, the facilitated charge transfer led to an enhanced efficiency. The insights gained from these observations may pave the way for a deeper exploration of POM structures, inspiring the development of novel approaches or designs that could potentially address the barriers to commercialization in PSCs.

## Data Availability

Data will be made available on request.

## Supporting Information Available

Some additional data including statistics, SEM pictures, hysteresis, and energy levels (PDF)
