# Supplementary material for: Polyoxometalate-Doped Hole Transport Layer to Boost Performance of MaPbI3-Based Inverted-Type Perovskite Solar Cells
Source: ACS Omega. 2025 Feb 14;10(7):6351–8. doi: 10.1021/acsomega.4c01242 (PMC11866188; doi:10.1021/acsomega.4c01242)
Supplement: Supplementary file 1 — ao4c01242_si_001.pdf [file ao4c01242_si_001.pdf]

Supporting Information

Polyoxometalate (POM) doped Hole Transport Layer to Boost Performance of $MaPbI_3$ Based Inverted Type Perovskite Solar Cells

Sumeyra Buyukcelebi[1], Mehmet Kazici[2], Yasemin Torlak[3], Mahmut Kus[4*], Mustafa Ersoz[5]

[1]Selcuk University, Advanced Technology Research and Application Center, Konya Turkey
[2]Siirt University, Engineering Faculty, Department of Electrical & Electronics Engineering, Siirt Turkey
[3]Pamukkale University, Cal Vocational High School, Denizli Turkey
[4]Konya Technical University, Department of Chemical Engineering, Konya Turkey
[5]Selcuk University, Department of Chemistry, Konya Turkey
***Corresponding authors:** mahmutkus1@gmail.com

Statistical calculations:

Oneway ANOVA with Tukey post hoc analysis was performed by using origin software. Statistical significance was based on the total error criterion at the 95.0% confidence level, therefore P values less than 0.05 were considered statistically significant. Twenty-four (24) independent electrical conductivity results for HTLs were compared using a statistical approach. All statistical results obtained from the one-way ANOVA with Tukey post hoc analysis are illustrated in Figure S1.

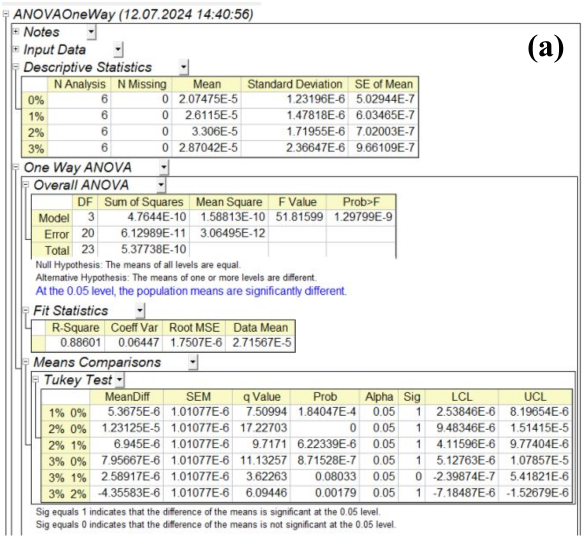
(a)

ANOVAOneWay (12.07.2024 14:40:56)
Notes
Input Data
Descriptive Statistics

	N Analysis	N Missing	Mean	Standard Deviation	SE of Mean
0%	6	0	2.07475E-5	1.23196E-6	5.02944E-7
1%	6	0	2.6115E-5	1.47818E-6	6.03465E-7
2%	6	0	3.306E-5	1.71955E-6	7.02003E-7
3%	6	0	2.87042E-5	2.36647E-6	9.66109E-7

One Way ANOVA
Overall ANOVA

	DF	Sum of Squares	Mean Square	F Value	Prob>F
Model	3	4.7644E-10	1.58813E-10	51.81599	1.29799E-9
Error	20	6.12989E-11	3.06495E-12		
Total	23	5.37738E-10			

Null Hypothesis: The means of all levels are equal.
Alternative Hypothesis: The means of one or more levels are different.
At the 0.05 level, the population means are significantly different.

Fit Statistics

	R-Square	Coeff Var	Root MSE	Data Mean
	0.88601	0.06447	1.7507E-6	2.71567E-5

Means Comparisons
Tukey Test

	MeanDiff	SEM	q Value	Prob	Alpha	Sig	LCL	UCL
1% 0%	5.3675E-6	1.01077E-6	7.50994	1.84047E-4	0.05	1	2.53846E-6	8.19654E-6
2% 0%	1.23125E-5	1.01077E-6	17.22703	0	0.05	1	9.48346E-6	1.51415E-5
2% 1%	6.945E-6	1.01077E-6	9.7171	6.22339E-6	0.05	1	4.11596E-6	9.77404E-6
3% 0%	7.95667E-6	1.01077E-6	11.13257	8.71528E-7	0.05	1	5.12763E-6	1.07857E-5
3% 1%	2.58917E-6	1.01077E-6	3.62263	0.08033	0.05	0	-2.39874E-7	5.41821E-6
3% 2%	-4.35583E-6	1.01077E-6	6.09446	0.00179	0.05	1	-7.18487E-6	-1.52679E-6

Sig equals 1 indicates that the difference of the means is significant at the 0.05 level.
Sig equals 0 indicates that the difference of the means is not significant at the 0.05 level.

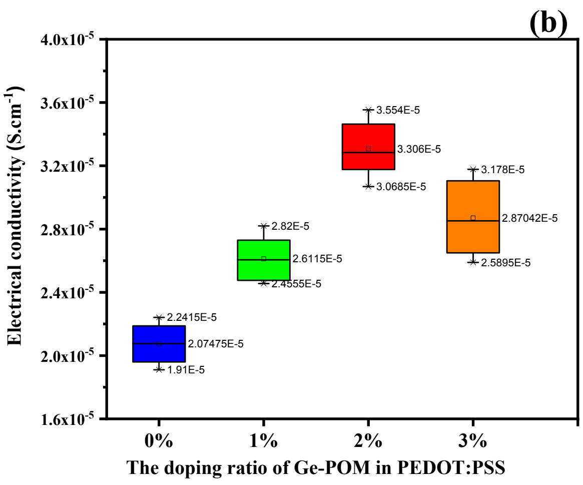

Figure S1. (a) Oneway ANOVA with Tukey post hoc analysis and (b) Twenty-four (24) independent electrical conductivity results for HTLs and their mean values.

The Oneway ANOVA analysis shows that there are different results between each categories (P value<< 0.5). According to the Tukey multiple comparison test, it was evident that the electrical conductivity of 2% GePOM-doped PEDOT:PSS was significantly higher than that of all other categories (see in figure 1a and 1b). All differences are statistically significant except for the 1% and 3% pair. Although the electrical conductivities of 1% and 3% GePOM-doped HTLs were better than that of the undoped (0%) HTLs, the Tukey comparison showed no significant difference between the electrical conductivities of the 1% and 3% GePOM-doped HTLs. Therefore, there was no need to investigate higher concentrations of GePOM in the HTL to further improve its electrical conductivity.

Energy levels:

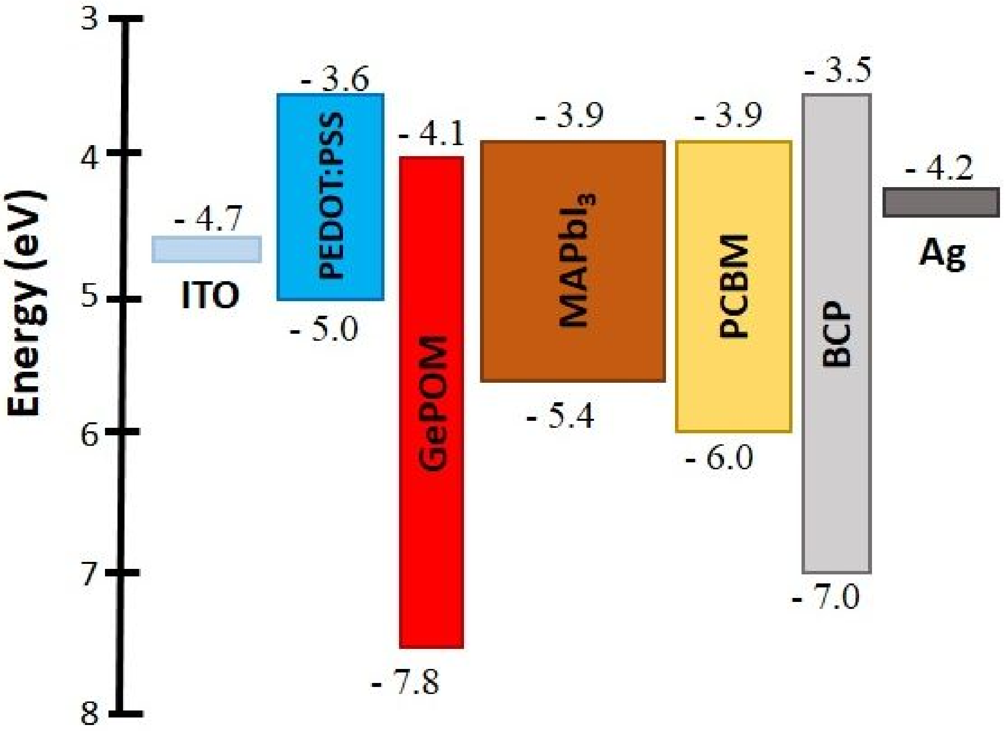

Figure S2. Energy levels of materials used in PSCs. Electrochemical method (cyclic volatmmetry technique) is used to calculate the energy levels of GePOM

SEM results:

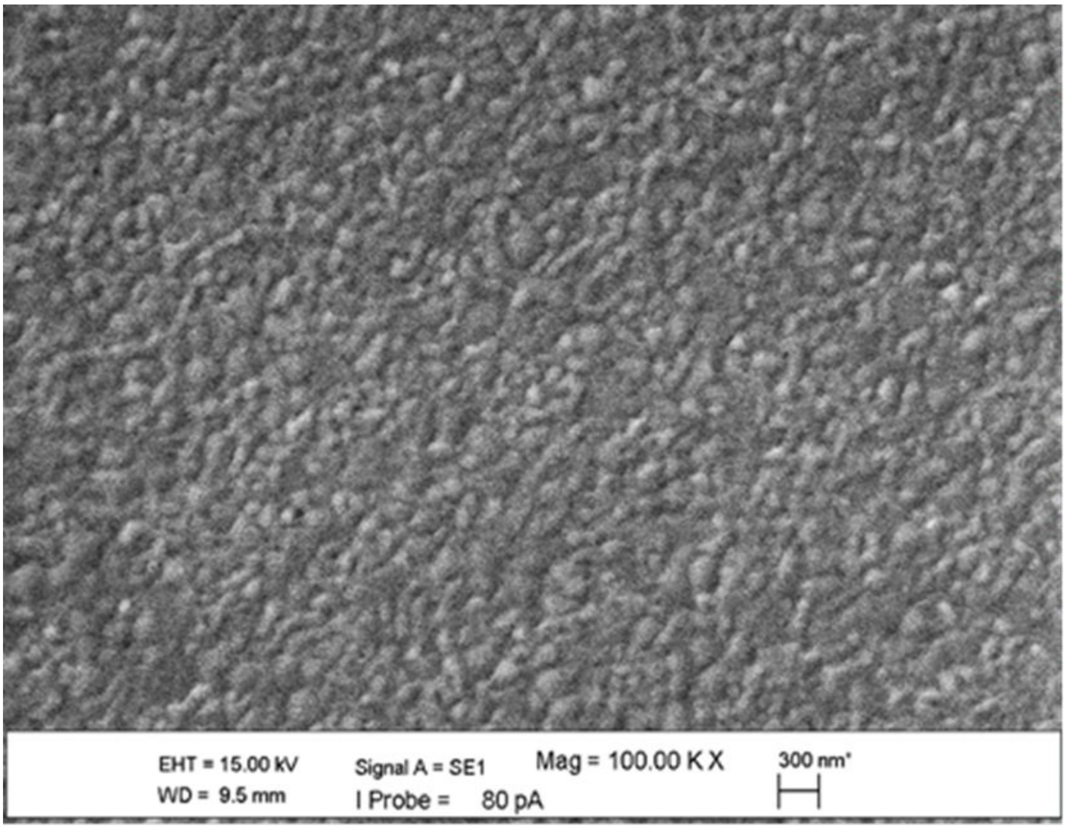

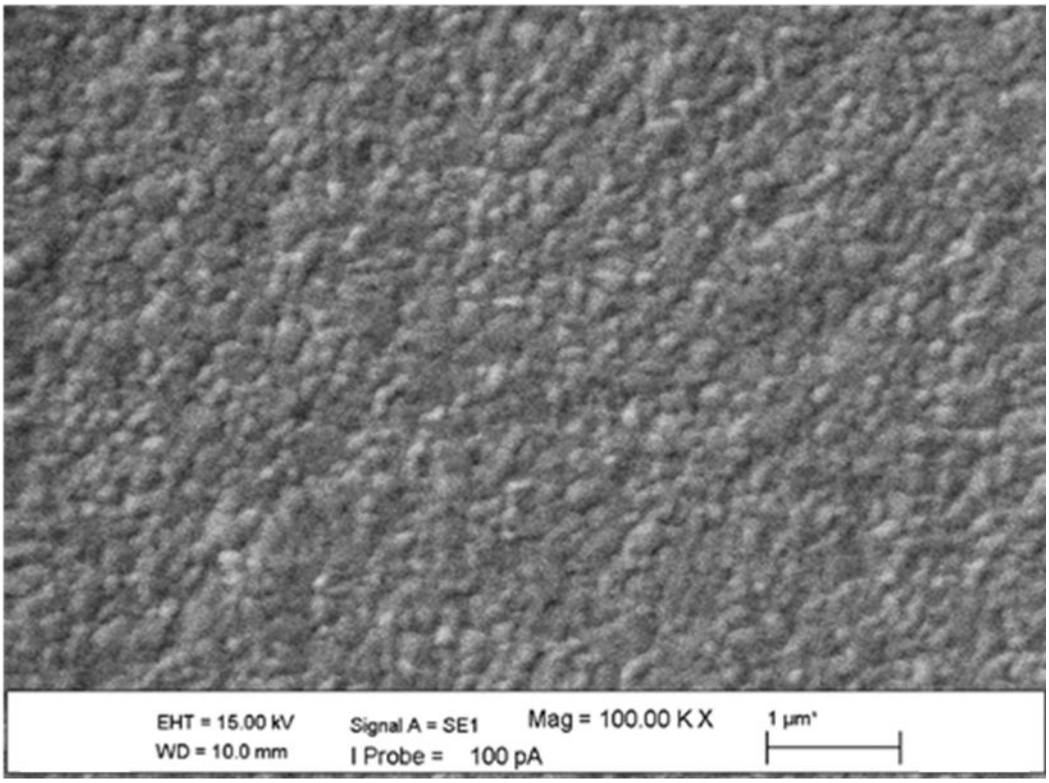

Figure S3. SEM images of perovskite surfaces. ITO/PEDOT:PSS/Perovskite is up and ITO/PEDOT:PSS:GePOM/Perovskite is down

Hysterises effect:

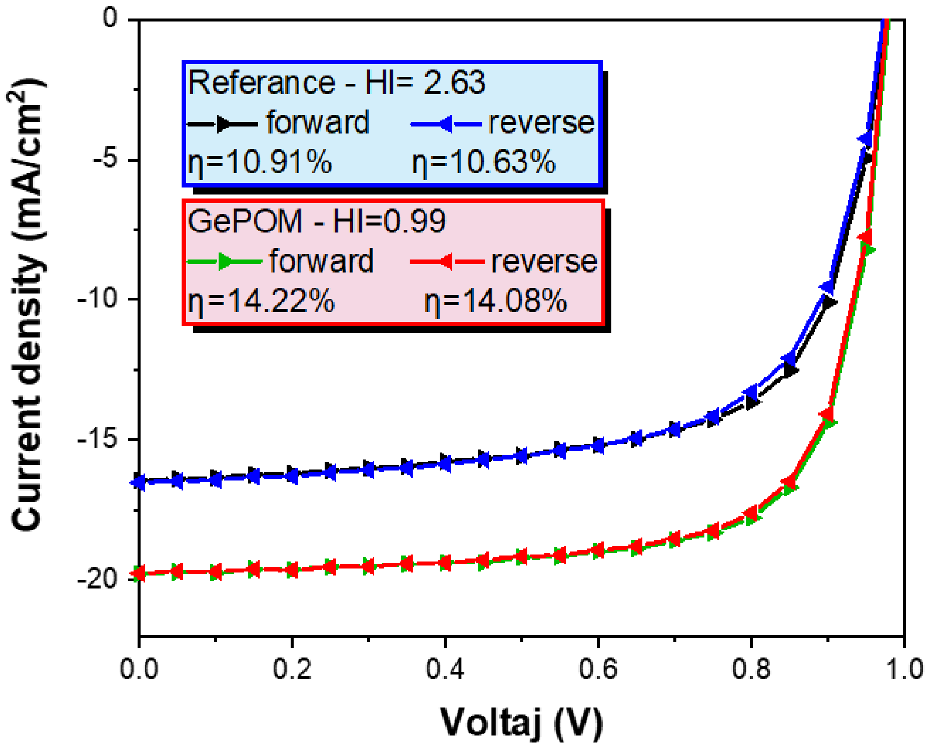

Figure S4. Hysterises effect of doped and undoped PSCs
